# Sleep quality of vulnerable elderly people: associated factors

**DOI:** 10.1590/0034-7167-2023-0283

**Published:** 2024-11-29

**Authors:** Ariene Angelini dos Santos-Orlandi, Ana Carolina Ottaviani, Elén dos Santos Alves, Tábatta Renata Pereira de Brito, Keika Inouye

**Affiliations:** I Universidade Federal de São Carlos. São Carlos, São Paulo, Brazil.; II Universidade Federal de Alfenas. Alfenas, Minas Gerias, Brazil.

**Keywords:** Geriatric Nursing, Aging, Frail Elderly, Sleep Quality, Social Vulnerability, Enfermería Geriátrica, Envejecimiento, Anciano Frágil, Calidad de Sueño, Vulnerabilidad Social

## Abstract

**Objective:**

To identify factors associated with poor sleep quality in elderly dependent individuals in social vulnerability.

**Method:**

Cross-sectional study with 59 elderly dependent individuals assisted by Family Health Units in São Carlos/SP. The following tools were used: Katz Index, Lawton and Brody Scale, Pittsburgh Sleep Quality Index, Addenbrooke’s Cognitive Examination Revised, Fried’s Frailty Phenotype, Geriatric Depression Scale (15 items), Perceived Stress Scale, Family APGAR, Social Support Scale from the Medical Outcomes Study, and World Health Organization Quality of Life, abbreviated and “old” versions.

**Results:**

The majority of participants were women (52.5%), aged 60-74 years (71.1%), and had poor sleep quality (76.2%). Stress (OR=1.12; 95%CI=1.02-1.22) and polypharmacy (OR=7.39; 95%CI=1.22-44.73) increased the chances of poor sleep quality, while physical activity decreased these chances (OR=0.15; 95%CI=0.02-0.79).

**Conclusion:**

Stress and polypharmacy are associated with poor sleep quality in elderly dependent individuals.

## RESUMEN

**Objetivo:**

Identificar factores asociados con la mala calidad del sueño en personas mayores dependientes en situación de vulnerabilidad social.

**Método:**

Estudio transversal, con 59 personas mayores dependientes asistidas por Unidades de Salud de la Familia de São Carlos/SP. Se utilizaron las siguientes herramientas: Escala de Katz, Escala de Lawton y Brody, Índice de Calidad del Sueño de Pittsburgh, Examen Cognitivo Revisado de Addenbrooke’s, Fenotipo de Fragilidad de Fried, Escala de Depresión Geriátrica (15 ítems), Escala de Estrés Percibido, APGAR de Familia, Escala de Apoyo Social del Medical Outcomes Study, y Calidad de Vida de la Organización Mundial de la Salud, versiones abreviada y “old”.

**Resultados:**

La mayoría de los participantes eran mujeres (52,5%), en el rango de edad de 60-74 años (71,1%), y presentaban mala calidad del sueño (76,2%). El estrés (OR=1,12; IC95%=1,02-1,22) y el uso de polifarmacia (OR=7,39; IC95%=1,22-44,73) aumentaron las probabilidades de mala calidad del sueño, mientras que la actividad física disminuyó dichas probabilidades (OR=0,15; IC95%=0,02-0,79).

**Conclusión:**

El estrés y la polifarmacia están asociados con la mala calidad del sueño en personas mayores dependientes.

## INTRODUCTION

In Brazil and worldwide, technological advances, reduced mortality rates, and declining birth rates are related to the increased life expectancy of the population^(1)^. Data from the Pan American Health Organization show that the global population is aging rapidly, especially in Latin America. In 2020, 8% of the population were elderly people, aged 60 or older, and it is estimated that by 2050 this percentage will double and, by the end of the century, exceed 30% of the world population^(2)^. Given the accelerated aging phenomenon and its various demands, it is important to observe not only the biological vulnerability resulting from aging but also to consider socioeconomic and psychosocial vulnerability^(3)^, as older people are more socially vulnerable^(3)^.

Social vulnerability can be understood as a set of resources involving housing context, education, financial assets, and access to opportunities that affect people’s quality of life^(4)^. The São Paulo Index of Social Vulnerability (IPVS) is an indicator that considers socioeconomic and demographic dimensions. It classifies the population of São Paulo into seven levels of vulnerability: Group 1 (very low vulnerability), Group 2 (low vulnerability), Group 3 (medium-low vulnerability), Group 4 (medium vulnerability), Group 5 (high vulnerability – urban sectors), Group 6 (very high vulnerability), and Group 7 (high vulnerability – rural sectors)^(5)^.

A systematic review conducted to identify risk and protective factors associated with frailty in community-dwelling elderly people indicated that living in high-density neighborhoods and the socioeconomic status of the neighborhood were positively associated with frailty conditions in the elderly. These data suggest that, beyond biological and psychological conditions, social components seem to play a significant role in the health conditions of elderly people over the years^(6)^. An integrative literature review aimed at understanding social vulnerability among the elderly population in Latin America demonstrated that the lack of public investment and social protection policies negatively impacts the lives and health of elderly people in social vulnerability^(4)^.

Sleep is an indispensable element for people’s health. It provides physical and mental rest, a sense of well-being, and energy recovery for performing daily tasks^(7)^. Changes in sleep architecture, circadian rhythm, and sleep patterns occur as people age, affecting sleep duration, quality, and timing^(8)^. Furthermore, poor sleep quality in the elderly is associated with highly prevalent chronic health conditions such as hypertension^(9)^, type 2 diabetes^(10)^, cardiovascular disease^(9)^, depression^(11)^, stress^(12)^, cognitive impairment^(13)^, and increased mortality risk^(8)^. The literature suggests that sociodemographic and lifestyle factors may play a role in sleep quality among the elderly. Factors such as female gender, low education, divorce and widowhood, living alone, inadequate fruit intake, drinking tea, alcohol consumption, caffeine intake, use of certain medications, falls, pain, and physical inactivity have been reported as associated with poor sleep quality^(12,13,14,15,16,17)^.

A study conducted in Brazil found that quality of life and social support are protective factors against depressive symptoms in elderly people living in socially vulnerable contexts^(18)^. However, sociodemographic, lifestyle, and health factors may differ across social contexts. Given the restriction of social, economic, and access to goods and services resources, there is a risk of illness and health-related damage in elderly people^(18)^. From this perspective, there may be a higher prevalence of poor sleep quality^(19)^.

Although studies on elderly dependent individuals in situations of high social vulnerability are scarce, the literature describes that socioeconomic conditions, particularly in the context of social vulnerability, are determinants of health^(4,17)^. Therefore, identifying potentially modifiable factors related to poor sleep quality can help develop effective interventions to mitigate sleep-related problems, as well as the risk of chronic conditions such as cardiovascular diseases, hypertension, diabetes mellitus, stress, and depression in dependent elderly individuals. It is anticipated that factors such as female gender, advanced age, low education, low income, presence of multimorbidities, depressive symptoms, stress, physical inactivity, pain, and functional impairment are associated with poor sleep quality in elderly people in contexts of high social vulnerability.

## OBJECTIVE

To identify factors associated with poor sleep quality in elderly dependent individuals in social vulnerability.

## METHODS

### Ethical Aspects

All ethical guidelines related to research involving human subjects were respected. The research was approved by the Research Ethics Committee of the Federal University of São Carlos, and the approval statement is attached to this submission. Participants were informed about the objectives, consulted regarding their availability to participate in the study, and assured of the confidentiality of their individual information. Data collection was conducted only after the signing of the Informed Consent Form.

### Design, Period, and Location of the Study

This is a cross-sectional, convenience quantitative study. All guidelines of the Strengthening the Reporting of Observational Studies in Epidemiology (STROBE) were followed. Data were collected from July 2019 to March 2020, with the assistance of five out of the nineteen Family Health Units (USFs) in the municipality of São Carlos, São Paulo, Brazil – a medium-sized municipality located in the central region of the state. These USFs were located in areas of high social vulnerability according to the criteria of the IPVS of the State System Data Analysis Foundation (SEADE).

### Sample, Inclusion, and Exclusion Criteria

Using a list of 168 names and addresses of potential participants provided by the USF health teams, the inclusion criteria were established: residing in an area covered by a USF in a context of social vulnerability in the municipality (IPVS 5), being 60 years or older, and being dependent on care for at least one basic activity of daily living assessed by the Katz Index (BADL) and/or instrumental activity of daily living assessed by the Lawton and Brody Scale (IADL). Through home visits, 59 elderly individuals were identified who met the inclusion criteria and showed interest and availability to participate in the research.

The exclusion criteria were: presenting hearing and/or speech deficits (stuttering, aphasia, and dysarthria) that could hinder the application and interpretation of the proposed instruments’results. The initial intention was to interview all elderly dependents on the list provided by the USFs; therefore, no sample calculation was performed. However, considering the population of 168 potential elderly dependents provided by the USFs, 59 participants represented a sample with a 95% confidence level and a 10% margin of error – numbers generated by the Survey Monkey® platform. Data collection was carried out at home, with written informed consent from the dependent elderly individuals.

### Study Protocol

With the list of potential participants, home visits were conducted by the researchers. Once the inclusion criteria were met, the research objectives were presented and, after obtaining consent and signing the Informed Consent Form, a new visit was scheduled according to the elderly individual’s availability for instrument application, lasting approximately one hour and thirty minutes. It is noteworthy that all researchers were undergraduate and graduate students, trained and qualified to apply the instruments.

The instruments used were:

- The Characterization Form for Dependent Elderly People was specifically developed for this study to describe the participant, their health context, and lifestyle habits. It requested information on: sex (male or female), age group (60 to 74 years or 75 years and older), race/color (white, black, brown, indigenous, or yellow), marital status (with a partner or without a partner), years of education (≤ 4 years or > 4 years), religion (Catholic, Evangelical, other, or none), current work (yes or no), retirement (yes or no), sufficient income (yes or no), number of people living in the household (≤ 2 or > 2), health insurance (yes or no), falls in the last year (yes or no), hospitalizations in the last year (yes or no), Body Mass Index (BMI) (underweight, normal weight, or overweight), physical activity (yes or no), pain (yes or no), multimorbidity (yes or no), polypharmacy (yes or no – use of five or more medications), smoking (yes or no), alcohol consumption (yes or no), coffee consumption (yes or no), green tea consumption (yes or no), mate tea consumption (yes or no), black tea consumption (yes or no), chocolate consumption (yes or no), ginger consumption (yes or no), pepper consumption (yes or no), and guarana consumption (yes or no).
- The Katz Index is an instrument used to assess dependence/ independence in basic activities of daily living (BADL), which includes areas of daily life such as bathing, dressing, using the bathroom, transferring, continence, and feeding. For each area, the instrument offers three response options: independent, needs assistance, and dependent. In the end, it is possible to verify in how many activities the elderly person is dependent^(20)^.
- The Lawton and Brody Scale was used to analyze dependence/ independence in instrumental activities of daily living (IADL) in areas such as the ability to use the telephone, prepare meals, perform housework, use medications, and manage money. This scale has a final score that can range from zero to 21 points, categorized as follows: a total of 7 points indicates total dependence, 8 to 20 points indicates partial dependence, and 21 points indicates independence^(21,22)^.
- The Pittsburgh Sleep Quality Index (PSQI) was used to assess sleep quality. It is composed of 19 self-reported questions. The instrument evaluates 7 components: subjective sleep quality, sleep latency, sleep duration, habitual sleep efficiency, sleep disturbances, use of sleep medication, and daytime dysfunction. The score can range from 0 to 21 points and can be categorized as good sleep quality (0 to 4 points), poor sleep quality (5 to 10 points), and the presence of sleep disturbances (11 to 21 points)^(23)^.
- The Addenbrooke’s Cognitive Examination Revised (ACER) was used for cognitive assessment, consisting of a brief cognitive evaluation battery. It comprises five cognitive domains with scores ranging from 0 to 100 points: Attention and Orientation (total score of 18 points); Memory (total score of 26 points); Fluency (total score of 14 points); Language (total score of 26 points); and Visuospatial abilities (total score of 16 points)^(24)^.
- Fried’s Frailty Phenotype was used for frailty classification. The instrument consists of five elements: unintentional weight loss, fatigue, low grip strength, low physical activity level, and slow walking speed. The presence of three or more of these characteristics can indicate that the elderly person is categorized as pre-frail, frail, or robust^(25)^.
- The Geriatric Depression Scale 15 (GDS-15) was used for screening depressive symptoms. It consists of a questionnaire with 15 questions, with yes or no answers, and a final score ranging from zero to 15 points, categorized as follows: from 0 to 5 points, no indication of depressive symptoms; from 6 to 10 points, mild depressive symptoms; and from 11 to 15 points, severe depressive symptoms^(26,27)^.
- The Perceived Stress Scale was used to quantify the level of stress. The scale has 14 questions with scores ranging from zero to 56 points. The total score indicates that the higher the final score, the greater the degree of stress perceived by the elderly person^(28)^.
- The Family APGAR was used to categorize family functionality. The instrument consists of 5 questions that measure satisfaction with the family in relation to adaptation, partnership, development, affection, and problem-solving capacity. The total score can range between zero and 20 points and is classified as follows: high family dysfunction (0-8 points), moderate family dysfunction (9-12 points), and good family functionality (13-20 points)^(29)^.
- For assessing quality of life, the World Health Organization Quality of Life scales, abbreviated version and version for the elderly (WHOQOL-bref and WHOQOL-old), were used. The abbreviated version has 26 items that evaluate four domains: physical, psychological, social relationships, and environment, as well as self-perception of quality of life and satisfaction with health. The higher the score, the better the elderly person’s perception of their quality of life^(30)^. The old version consists of 24 questions that assess six domains: sensory abilities, autonomy, past, present, and future activities, social participation, death and dying, and intimacy. The total score ranges from four to 20 points, and the higher the final score, the better the elderly person’s perception of their quality of life^(31)^.
- The Medical Outcomes Study Social Support Scale is an instrument used to analyze the availability of material, emotional, affectionate, informational, and positive social interaction support. It is composed of 19 items with scores ranging from 20 to 100 points, where the higher the score, the greater the level of social support for the individual^(32)^. The instruments used for data collection were translated and validated for use in the Brazilian context.

### Analysis of Results and Statistics

The data obtained were coded and entered into an electronic spreadsheet and analyzed using the Stata statistical package, version 13. Frequency distributions, means, standard deviations, medians, 25th percentile (p25), and 75th percentile (p75) were estimated for numerical variables, and proportions were estimated for categorical variables. The Kolmogorov-Smirnov test was used to test the normality of the variables. Differences between groups for normally distributed variables were calculated using the t-test, and for non-normally distributed variables, the Mann-Whitney test was used. For categorical variables, Pearson’s chi-square test and Fisher’s exact test were used.

For association analysis, multiple logistic regression was used, with the magnitude of the association estimated by the crude and adjusted odds ratio (OR). Variables with a p-value less than 0.20 in the univariate analysis were included in the multiple model using the stepwise forward procedure. The final model included variables that showed statistical significance (p<0.05) and the variables sex and age group for adjustment. A significance level of 5% was used in all analyses.

## RESULTS

Table 1 presents the sociodemographic characteristics of the participants according to poor sleep quality in detail. The majority of the dependent elderly were female (52.54%) and in the age group of 60 to 74 years (71.19%). No statistically significant differences were found between the sociodemographic characteristics and poor sleep quality. Approximately 76.27% of the participants had poor sleep quality.

**Table 1:** Sociodemographic characteristics of dependent elderly people according to poor sleep quality. São Carlos, São Paulo, Brazil, 2019-2020

Variable	Total n(%)	Poor Sleep Quality			OR(95%IC)
		Yes n(%)	No n(%)	*p* value	
Sex					
Male	28(47.46)	9(32.14)	19(67.86)	0.149^1^	
Female	31(52.54)	5(16.13)	26(83.87)		2.46(0.71-853)
Age Group					
60 to 74 years	42(71.19)	10(23.81)	32(76.19)	1.000^2^	
75 years or older	17(28.81)	4(23.53)	13(76.47)		1.01(0.26-3.82)
Race/Color					
White	22(37.29)	6(27.27)	16(72.73)	0.835^2^	
Black	11(18.64)	3(27.27)	8(72.73)		1.00(0.19-5.07)
Brown	25(42.37)	5(20.00)	20(80.00)		1.50(0.38-5.82)
Indigenous	0(0.00)	0(0.00)	0(0.00)		-
Yellow	1(1.69)	0(0.00)	1(100.00)		-
Marital Status					
With partner	54(91.53)	13(24.07)	41(75.93)	1.000^2^	
Without partner	5(8.47)	1(20.00)	4(80.00)		1.26(1.29-12.37)
Years of Education					
≤ 4 years	49(83.05)	11(22.45)	38(77.55)	0.688^2^	
4 years	10(16.95)	3(30.00)	7(70.00)		0.67(0.14-3.05)
Religion					
Catholic	33(55.93)	8(24.24)	25(75.76)	0.276^2^	
Evangelical	17(28.81)	6(35.29)	11(64.71)		0.58(0.16-2.09)
Other	7(11.86)	0(0.00)	7(100.00)		-
None	2(3.39)	0(0.00)	2(100.00)		-
Currently Working					
No	57(96.61)	14(24.56)	43(75.44)	1.000^2^	
Yes	2(3.39)	0(0.00)	2(100.00)		-
Retired					
No	8(13.56)	0(0.00)	8(100.00)	0.179^2^	
Yes	51(86.44)	14(27.45)	37(72.55)		-
Sufficient Income No	33(55.93)	5(15.15)	28(84.85)	0.081^2^	
Yes	26(44.07)	9(34.62)	17(65.38)		0.33(0.09-1.17)
Number of Household Members					
≤ 2 people	35(59.32)	7(20.00)	28(80.00)	0.416^1^	
2 people	24(40.68)	7(29.17)	17(70.83)		0.60(0.18-2.03)
Family Functionality*					
Functional	38(67.86)	7(18.42)	31(81.58)	0.217^1^	
Dysfunctional	18(32.14)	6(33.33)	12(66.67)		0.45(0.12-1.62)
Total	59(100.00)	14(23.73)	45(76.27)		

^1^Pearson’s Chi-square Test; ^2^Fisher’s Exact Test; *some participants did not respond; OR: Odds ratio.

No statistically significant differences were found between the sociodemographic characteristics and poor sleep quality. Table 2 presents the detailed health characteristics of dependent elderly people according to poor sleep quality.

**Table 2:** Health characteristics of dependent elderly people according to poor sleep quality. São Carlos, São Paulo, Brazil, 2019-2020

Variable	Total n(%)	Poor Sleep Quality			OR(95%IC)
		Yes n(%)	No n(%)	*p* value	
Health Insurance					
No	53(89.83)	11(20.75)	42(79.25)	0.139^2^	
Yes	6(10.17)	3(50.00)	3(50.00)		0.26(0.04-1.48)
Fall in the Last Year					
No	41(69.49)	12(29.27)	29(70.73)	0.189^2^	
Yes	18(30.51)	2(11.11)	16(88.89)		3.31(0.65-16.67)
Hospitalization in the Last Year					
No	49(83.05)	13(26.53)	36(73.47)	0.425^2^	
Yes	10(16.95)	1(10.00)	9(90.00)		3.25(0.37-28.21)
BMI*					
Underweight	6(10.71)	1(16.67)	5(83.33)	0.905^2^	
Normal weight	17(30.36)	5(29.41)	12(70.59)		0.48(0.04-5.22)
Overweight	33(58.93)	8(24.24)	25(75.76)		0.62(0.63-6.17)
Physical Activity					
No	41(69.49)	7(17.07)	34(82.93)	0.070^1^	
Yes	18(30.51)	7(38.89)	11(61.11)		0.32(0.09-1.12)
Pain*					
No	13(22.81)	3(23.08)	10(76.92)	1.000^2^	
Yes	44(77.19)	10(22.73)	34(77.27)		1.02(0.23-4.43)
Multimorbidity					
No	1(1.69)	1(100.00)	0(0.00)	0.237^2^	
Yes	58(98.31)	13(22.41)	45(77.59)		-
Polypharmacy*					
No	28(48.28)	10(35.71)	18(64.29)	0.067^2^	
Yes	30(51.72)	4(13.33)	26(86.67)		3.61(0.97-13.33)
Frailty*					
No	29(50.88)	10(34.48)	19(65.52)	0.123^2^	
Yes	28(49.12)	4(14.29)	24(85.71)		
Depressive Symptoms*					
No	28(50.00)	9(32.14)	19(67.86)	0.205^2^	
Yes	28(50.00)	4(14.29)	24(85.71)		2.84(0.75-10.66)
Cognitive Decline					
No	11(18.64)	1(9.09)	10(90.91)	0.269^2^	
Yes	48(81.36)	13(27.08)	35(72.92)		0.26(0.03-2.31)
BADL					
Independent	30(50.85)	7(23.33)	23(76.67)	0.942^1^	
Dependent	29(49.15)	7(24.14)	22(75.86)		0.95(0.28-3.17)
IADL					
Independent	1(1.69)	0(0.00)	1(100.00)	1.000^2^	
Dependent	58(98.31)	14(24.14)	44(75.86)		-
Smoking					
No	51(86.44)	14(27.45)	37(72.55)	0.179^2^	
Yes	8(13.56)	0(0.00)	8(100.00)		-
Alcohol Consumption					
No	51(86.44)	12(23.53)	39(76.47)	1.000^2^	
Yes	8(13.56)	2(25.00)	6(75.00)		0.92(0.16-5.18)

^1^Pearson's Chi-square Test; ^2^Fisher's Exact Test; *some participants did not respond; OR: Odds ratio; BMI: Body Mass Index; BADL: Basic Activities of Daily Living; IADL: Instrumental Activities of Daily Living.

No statistically significant differences were found between health characteristics and poor sleep quality.

The majority of elderly people consumed coffee (84.75%) and did not consume green tea (76.27%), mate tea (71.19%), black tea (91.53%), chocolate (72.88%), ginger (77.97%), peppers (64.41%), or guarana (98.31%). No statistically significant differences were found between the groups (good/poor sleep quality) regarding the consumption of these beverages and foods.

Table 3 presents the relationship between stress, quality of life, and social support variables according to poor sleep quality in dependent elderly people.

**Table 3:** Comparative analysis of stress, quality of life, and social support variables according to poor sleep quality in dependent elderly people. São Carlos, São Paulo, Brazil, 2019-2020

Variable	Poor Sleep Quality				*p* value
	No		Yes		
	Mean (SD)	Median (p25;p75)	Mean (SD)	Median (p25;p75)	
Stress	18.00 (10.16)	17.00 (13;27)	24.86 (10.93)	24.00 (19;33)	0.041^1^
Quality of Life	69.40 (22.02)	72.08 (64.16;84.16)	66.00 (17.35)	68.33 (64.16;76.66)	0.268^2^
Material Support	85.71 (27.44)	100 (80;100)	87.44 (23.27)	100 (85;100)	0.884^2^
Affective Support	86.19 (26.20)	96.66 (80;100)	84.59 (26.62)	100 (86.66;100)	0.845^2^
Emotional Support	75.71 (26.80)	80 (65;100)	76.55 (27.54)	90 (65;100)	0.758^2^
Positive Social Interaction	77.85 (26.21)	80 (70;100)	71.11 (27.77)	75 (60;100)	0.361^2^
Information	78.92 (26.54)	85 (70;100)	74.22 (28.02)	85 (50;100)	0.683^2^

*^1^ t-test; ^2^ Mann-Whitney test.*

A statistically significant relationship was observed between stress and poor sleep quality. Dependent elderly people with poor sleep quality had higher stress scores compared to those with good sleep quality.

In the multiple logistic regression model, stress (OR=1.12; 95% CI=1.02-1.22) and polypharmacy (OR=7.39; 95% CI=1.22-44.73) increased the chances of poor sleep quality in dependent elderly people, while physical activity reduced these chances (OR=0.15; 95% CI=0.02-0.79), independent of sex and age group, as shown in Table 4. Table 4 presents the factors associated with poor sleep quality.

**Table 4:** Factors associated with poor sleep quality in dependent elderly people. São Carlos, São Paulo, Brazil, 2019-2020

	OR_adj_^1^	*p* value	95% CI
Stress	1.12	0.017	1.02-1.22
Physical Activity			
No	1.00		
Yes	0.15	0.026	0.02-0.79
Polypharmacy			
No	1.00		
Yes	7.39	0.029	1.22-44.73
Sex			
Male	1.00		
Female	0.77	0.756	0.15-3.79
Age Group			
60 to 74 years	1.00		
75 years or older	1.38	0.688	0.28-6.74

*^1^OR_adj_= Adjusted Odds Ratio; 95% CI: 95% Confidence Interval.*

## DISCUSSION

This study identified factors associated with poor sleep quality in elderly dependent individuals living in socially vulnerable areas. Factors such as stress and polypharmacy increased the likelihood of poor sleep quality, while physical activity decreased these chances, regardless of sex and age group.

In the present study, there was a predominance of poor sleep quality among dependent elderly individuals. National and international research corroborated these findings, also identifying high proportions of complaints related to nighttime sleep^(33,34,35)^.

Evidence in the literature indicates that some changes that occur in individuals’bodies during the aging process can interfere with both the quantity and quality of nighttime sleep. Factors such as reduced melatonin, circadian rhythm modifications, neuroendocrine dysfunctions, medical comorbidities, decreased social involvement, and environmental disturbances contribute to impaired nighttime sleep^(36,37)^. Additionally, evidence shows that family factors and social conditions can affect sleep quality^(38)^.

In the present study, there was an association between poor sleep quality and stress. A study conducted in India aimed at identifying the prevalence of poor sleep quality and its associated factors observed 180 elderly individuals. The results showed that 68.9% of the sample had poor sleep quality, and the factors associated with poor sleep were depression, anxiety, and not having a partner^(35)^.

Corroborating these findings, research conducted in the USA with 4,201 people aged 65 and older found that those who were highly stressed had almost five times the likelihood of having poor sleep quality compared to those with low stress levels^(12)^.

Chinese researchers conducted a study with elderly individuals and found a significant correlation between sleep quality and psychological distress, including stress. They also observed that poor sleep quality can worsen mood and increase anxiety, generating high stress levels throughout the day^(39)^.

For those with functional impairment dependent on daily activities, high levels of anxiety and depression were observed, which could lead to intense stress and poor sleep quality^(39)^. Often, because they cannot meet their needs independently, these individuals may feel useless, which affects mental health and impairs nighttime sleep^(39)^. Although this refers to a province comprising 11 cities in Central China, it is possible that this reasoning partly explains the association identified between stress and poor sleep quality in the present study, considering that there was a higher prevalence of poor sleep quality among dependent elderly individuals, both for BADL and IADL. On the other hand, experiencing poor nighttime sleep (whether due to difficulty falling asleep and staying asleep, or due to short sleep duration) can negatively impact the following day, such as worsening mood, increasing anxiety, and causing daytime fatigue, which could generate stress^(39)^.

In an attempt to reduce stress and, consequently, improve nighttime sleep quality, a randomized clinical trial with elderly individuals tested the effects of mindfulness over 8 weeks with a 6-month follow-up. The results showed that stress reduction based on mindfulness was effective, improving sleep quality in those with moderate to high levels of sleep disturbances. The study concluded that mind-body interventions are relevant in addressing factors associated with poor sleep quality^(40)^.

Under stress-inducing conditions, researchers state that cortisol is released to adapt to environmental demands. However, prolonged secretion of stress hormones can harm the individual by increasing chronic inflammation, reducing immunity, causing homeostatic imbalance, and inhibiting melatonin release, which would impair nighttime sleep^(41)^.

Among dependent elderly individuals, the use of polypharmacy increased the chances of poor sleep quality by about 6.39 times. The number of medications used was also related to sleep complaints in recent review studies identified in the international literature^(42)^.

Researchers point out that several mechanisms may explain the impairment of nighttime sleep in elderly people who use polypharmacy due to the influence exerted on receptors and neurotransmitters. Daytime sleepiness (with the use of antihistamines, anticonvulsants, opioids, for example), stimulating effects (with the use of beta agonists, pseudoephedrine, selegiline, for example), the potential to worsen existing sleep disorders (with the use of antidepressants, opioids, benzodiazepines, for example), the ability to impair sleep architecture by reducing melatonin secretion (beta-blockers, for example), drug-drug and/or drugdisease interactions that may cause nighttime sleep discomfort (nocturia, cough, hypoglycemia, for example), and side effects are factors that can explain the relationship found between polypharmacy and poor sleep^(42,43)^.

The results of the present research showed that physical activity was identified as a protective factor against poor sleep. Convergent findings were identified in the literature^(44,45)^. A literature review conducted with randomized clinical trials, quasi-experimental, and descriptive studies in Spain observed that physical exercise had positive effects (p<0.05) on improving sleep quality, especially in people over 55 years old and women^(44)^.

To investigate the effect of physical exercise on sleep quality and inflammatory cytokines in elderly individuals, a randomized clinical trial was conducted with 50 participants, who were randomly divided into two groups (A: intervention group with supervised aerobic exercises three times a week for six months, and B: control group, without intervention, following their normal lifestyle). The results showed that aerobic training reduced pro-inflammatory cytokine levels and improved nighttime sleep quality^(45)^.

Although physical activity and physical exercise are not synonymous, these terms have been used interchangeably in the literature, which has shown promising results for sleep quality when performed regularly^(44)^.

A recent review study showed that physical exercise has a beneficial effect on nighttime sleep quality in community-dwelling elderly individuals. The results indicated that aerobic exercises, of low to moderate intensity, for 30 minutes daily, three to five times a week, can result in good nighttime sleep quality. The study concluded that physical activity combined with sleep hygiene acts as a protective factor against sleep disorders^(44)^.

These researchers pointed out that the mechanism related to the positive effect of physical exercise on sleep quality is not yet clearly defined. It is believed that the secretion of endorphins and the increase in energy expenditure would facilitate restful sleep and, consequently, body recovery. Additionally, the reduction of low-grade inflammation would also reflect beneficial effects on sleep quality^(45)^.

Other scholars indicate that regular physical activity can improve the overall health of elderly individuals, with the beneficial impact of adopting a healthy lifestyle being notable. Physical exercise can strengthen functional abilities as well as delay or neutralize functional decline, contributing to improved quality of life and reduced psychological distress^(39)^.

Given the context of social vulnerability, characterized by scarce social or health resources, the assistance provided within the scope of Primary Health Care (PHC) is essential to offer care based on assertive and qualified actions, fundamentally grounded in understanding the needs of the elderly, considering the specificities of aging, and especially centered on comprehensive care for the individual, family, and community^(46)^.

### Limitations of the Study

The results of this study are subject to limitations. Given the cross-sectional design and the small, specific sample, there are limitations in terms of generalization and inference of causality. Additionally, the explanatory power of the models used in this study was low to moderate, even after including a significant number of potentially explanatory variables. Future research, particularly longitudinal studies, is necessary to deepen the understanding of this topic.

### Contributions to Nursing

The findings of the present study have potential implications for the development of policies and interventions that can contribute to health care, fostering recommendations to reduce poor sleep quality in this population. Additionally, the results can guide the planning of nursing actions and health care, considering the factors associated with poor sleep quality and the variables that can minimize it, as well as the social context. This is important because poor sleep quality can harm the health, quality of life, and well-being of the elderly.

## CONCLUSION

The hypothesis was partially confirmed. Stress and polypharmacy increased the chances of poor sleep quality, while physical activity reduced this chance.
