# Supplementary material for: Sleep quality of vulnerable elderly people: associated factors
Source: Rev Bras Enferm. 2024 Nov 29;77(Suppl 3):e20230283. doi: 10.1590/0034-7167-2023-0283 (PMC11654265; doi:10.1590/0034-7167-2023-0283)
Supplement: Translation

# Translation: Qualidade do sono de pessoas idosas vulneráveis: fatores associados

**Authors:** Ariene Angelini dos Santos-Orlandi, Ana Carolina Ottaviani, Elén dos Santos Alves, Tábatta Renata Pereira de Brito, Keika Inouye

**Affiliations:** I Universidade Federal de São Carlos. São Carlos, São Paulo, Brasil.; II Universidade Federal de Alfenas. Alfenas, Minas Gerias, Brasil.

**Keywords:** Enfermagem Geriátrica, Envelhecimento, Idoso Fragilizado, Qualidade do Sono, Vulnerabilidade Social

## Abstract

**Objetivo:**

Identificar fatores associados à má qualidade do sono em pessoas idosas dependentes em vulnerabilidade social.

**Método:**

Estudo transversal, com 59 pessoas idosas dependentes assistidas por Unidades de Saúde da Família de São Carlos/SP. Foram utilizadas as seguintes ferramentas: Escala de Katz, Escala de Lawton e Brody, Índice de Qualidade do Sono de Pittsburgh, Addenbrooke’s Cognitive Examination Revised, Fenótipo de Fragilidade de Fried, Escala de Depressão Geriátrica (15 itens), Escala de Estresse Percebido, APGAR de Família, Escala de Apoio Social do *Medical Outcomes Study*, e *World Health Organization Quality of Life*, versões abreviada e“*old*”.

**Resultados:**

A maioria dos participantes era composta por mulheres (52,5%), na faixa etária de 60-74 anos (71,1%), e apresentava má qualidade do sono (76,2%). O estresse (OR=1,12; IC95%=1,02-1,22) e o uso de polifarmácia (OR=7,39; IC95%=1,22-44,73) aumentaram as chances de má qualidade do sono, enquanto a atividade física diminuiu essas chances (OR=0,15; IC95%=0,02-0,79).

**Conclusão:**

Estresse e polifarmácia estão associados à má qualidade do sono de pessoas idosas dependentes.

## INTRODUÇÃO

No Brasil e no mundo, os avanços tecnológicos, a redução das taxas de mortalidade, e a queda das taxas de natalidade estão relacionadas ao aumento da expectativa de vida da população^(1)^. Dados da Organização Pan-Americana de saúde demonstram que a população mundial está envelhecendo de forma acelerada, especialmente na américa Latina. Em 2020, 8% da população eram pessoas idosas, com 60 anos ou mais, estima-se que até o ano de 2050 este percentual dobre e, até o final do século, exceda 30% da população mundial^(2)^. Em razão do fenômeno acelerado de envelhecimento e de suas diversas demandas, é importante observar não somente a vulnerabilidade biológica proveniente da senescência, mas considerar a vulnerabilidade socioeconômica e psicossocial^(3)^, ume vez que pessoas mais velhas são socialmente mais vulneráveis^(3)^.

A vulnerabilidade social pode ser compreendida como um conjunto de recursos que envolvem contexto de moradia, educação, posse financeira e acesso a oportunidades que interferem na qualidade de vida das pessoas^(4)^. O Índice Paulista de Vulnerabilidade Social (IPVS) é um indicador, no qual considera dimensões socioeconômicas e demográficas. Este classifica a população paulista em 7 níveis de vulnerabilidade, sendo Grupo 1 (baixíssima vulnerabilidade), Grupo 2 (vulnerabilidade muito baixa), Grupo 3 (vulnerabilidade baixa), Grupo 4 (vulnerabilidade média), Grupo 5 (vulnerabilidade alta – setores urbanos), Grupo 6 (vulnerabilidade muito alta) e Grupo 7 (vulnerabilidade alta – setores rurais)^(5)^.

Uma revisão sistemática realizada com o objetivo de identificar os fatores de risco e protetores associados a fragilidade em pessoas idosas da comunidade apontou que residir em bairros de alta densidade e o status socioeconômicos do bairro eram positivamente associados a condições de fragilidade das pessoas idosas. Estes dados apontam que para além das condições biológicos e psíquicas, os componentes socias parecem desempenhar um papel significativo nas condições de saúde de pessoas idosos ao longo dos anos^(6)^. Uma revisão integrativa de literatura, com o objetivo de compreender a questão da vulnerabilidade social na população idosa da América Latina, demonstrou que a escassez de investimentos públicos e em políticas de proteção social impactam de forma negativa na vida e na saúde de pessoas idosas em situação de vulnerabilidade social^(4)^.

O sono é um elemento indispensável para a saúde das pessoas. O sono propicia descanso físico e mental, sensação de bem estar, além da recuperação da energia para a execução das tarefas do cotidiano^(7)^. Alterações na arquitetura, ritmo circadiano e no padrão do sono ocorrem à medida que as pessoas envelhecem, repercutindo na duração, qualidade e tempo do sono^(8)^. Ademais, a má qualidade do sono em pessoas idosas está associada a condições crônicas de saúde altamente prevalentes, como hipertensão arterial^(9)^, diabetes tipo 2^(10)^, doença cardiovascular^(9)^, depressão^(11)^, estresse^(12)^, comprometimento cognitivo^(13)^ e risco aumentando para mortalidade^(8)^. A literatura sugere que os fatores sociodemográficos e de estilo de vida podem desempenhar um papel na qualidade do sono entre pessoas idosas, tais como sexo feminino, baixa escolaridade, divórcio e viuvez, morar sozinho, ingestão inadequada de frutas, beber chá, consumo de álcool, cafeína, uso de certos medicamentos, quedas, dor e inatividade física foram relatados como associados à má qualidade do sono^(12,13,14,15,16,17)^.

Um estudo realizado no Brasil constatou que a qualidade de vida e o suporte social são fatores protetores para sintomas depressivos em pessoas idosas inseridas em contextos vulneráveis socialmente^(18)^. No entanto, os fatores sociodemográficos, de estilo de vida e de saúde podem diferir entre os contextos sociais. Diante da restrição de recursos sociais, econômicos e de acesso a bens e serviços, existe o risco de adoecimento e de danos relacionados à saúde em pessoas idosas^(18)^. Nessa perspectiva, pode haver maior prevalência de má qualidade do sono^(19)^.

Embora os estudos com pessoas idosas dependentes em situação de alta vulnerabilidade social sejam escassos. De acordo com o descrito na literatura, as condições socioeconômicas, no que tange o contexto de vulnerabilidade social, são condições determinantes de saúde^(4,17)^. Diante disso, a identificação de fatores potencialmente modificáveis relacionados à má qualidade do sono pode ajudar a desenvolver intervenções eficazes para mitigar problemas relacionados ao sono, bem como, o risco de condições crônicas como doenças cardiovasculares, hipertensão, diabetes mellitus, estresse, depressão em pessoas idosas dependentes. Prospecta-se que fatores, como sexo feminino, idade avançada, baixa escolaridade, baixa renda, presença de multimorbidades, sintomas depressivos, estresse, inatividade física, dor e comprometimento funcional estejam associados à má qualidade do sono de pessoas idosas inseridos em contexto de alta vulnerabilidade social.

## OBJETIVO

Identificar fatores associados à má qualidade do sono em pessoas idosas dependentes em vulnerabilidade social.

## MÉTODOS

### Aspectos éticos

Todos as diretrizes éticas relativas às pesquisas com seres humanos foram respeitadas. A pesquisa foi aprovada pelo Comitê de Ética em Pesquisa da Universidade Federal de São Carlos, cujo parecer está anexado à presente submissão. Os participantes foram informados dos objetivos, consultados sobre a disponibilidade em participar do estudo e assegurados do sigilo das informações individuais. A coleta de dados foi realizada somente após assinatura do Termo de Consentimento Livre e Esclarecido.

### Desenho, período e local do estudo

Esta é uma pesquisa transversal e por conveniência do tipo quantitativo. Todas as diretrizes do *Strengthening the Reporting of Observational Studies in Epidemiology* – STROBE, foram atendidas. Os dados foram coletados, de julho de 2019 a março 2020, com o auxílio de cinco das dezenove das Unidades de Saúde da Família (USF) do município de São Carlos, São Paulo, Brasil – um município de porte médio localizado na região central do estado. Estas USF estavam localizadas em áreas de alta vulnerabilidade social segundo os critérios do Índice Paulista de Vulnerabilidade Social (IPVS) da Fundação Sistema Estadual de Análise de Dados Estatísticos – SEADE.

### Amostra, critérios de inclusão e exclusão

Por meio de uma lista com 168 nomes e endereços de potenciais participantes fornecida pelas equipes de saúde das USFs, foram estabelecidos os critérios de inclusão: residir em área de abrangência de uma USF inserida em contexto de vulnerabilidade social do município (IPVS 5), possuir 60 anos ou mais e ser dependente de cuidados em pelo menos uma atividade básica de vida avaliada pelo Índice de Katz (ABVD) e/ou instrumental de vida diária avaliada pela Escala de Lawton e Brody (AIVD). Por meio de visitas domiciliares, foram identificadas 59 pessoas idosas que atendiam aos critérios de inclusão e demonstraram interesse e disponibilidade para participar da pesquisa.

Os critérios de exclusão foram: apresentar déficits de audição e/ou linguagem (gaguez, afasia e disartria) que pudessem impossibilitar a aplicação e interpretação dos resultados dos instrumentos propostos. A intenção inicial era entrevistar todas as pessoas idosas dependentes da lista fornecida pelas USFs, por isso não foi feito um cálculo amostral. No entanto, ao considerar a população de 168 potenciais pessoas idosas dependentes fornecida pelas USFs, 59 participantes representaram uma amostra com nível de confiança de 95% e margem de erro de 10% – números gerados pela plataforma Survey Monkey®. A coleta de dados foi realizada nos domicílios, com consentimento documental escrito das pessoas idosas dependentes.

### Protocolo do estudo

De posse da lista de potenciais participantes, foram realizadas visitas domiciliares pelos pesquisadores. Atendidos os critérios de inclusão, os objetivos da pesquisa eram apresentados e, após o aceite e a assinatura do Termo de Consentimento Livre e Esclarecido, uma nova visita era agendada de acordo com a disponibilidade da pessoa idosa para aplicação dos instrumentos, com duração de aproximadamente uma hora e trinta minutos. Cabe salientar que todos os pesquisadores eram estudantes de graduação e pós-graduação, treinados e habilitados para aplicação dos instrumentos.

Os instrumentos utilizados foram:

- Ficha de Caracterização das Pessoas Idosas Dependentes para descrição do participante, de seu contexto de saúde e hábitos de vida, elaborada especificamente para este estudo, solicitava informações sobre: sexo (masculino ou feminino), faixa etária (60 a 74 anos ou 75 anos e mais), raça/cor (branca, preta, parda, indígena ou amarela), situação conjugal (com companheiro ou sem companheiro), anos de estudo (≤ 4 anos ou > 4 anos), religião (católica, evangélica, outra ou não possui), trabalho atual (sim ou não), aposentadoria (sim ou não), renda suficiente (sim ou não), número de pessoas que moram na casa (≤ 2 ou > 2), plano de saúde (sim ou não), quedas no último ano (sim ou não), internações no último ano (sim ou não), Índice de Massa Corporal (IMC) (baixo peso, eutrófico ou sobrepeso), prática de atividade física (sim ou não), dor (sim ou não), multimorbidade (sim ou não), polifarmácia (sim ou não – em uso de cinco ou mais medicamentos), tabagismo (sim ou não), etilismo (sim ou não), consumo de café (sim ou não), consumo de chá verde (sim ou não), consumo de chá mate (sim ou não), consumo de chá preto (sim ou não), consumo de chocolate (sim ou não), consumo de gengibre (sim ou não), consumo de pimenta (sim ou não), consumo de guaraná (sim ou não).
- O Índice de Katz é um instrumento utilizado para avaliar a dependência/independência em atividades básicas de vida diária (ABVD), que compreende áreas da vida cotidiana como banho, capacidade para se vestir, usar o banheiro, transferência, continência e alimentação. Para cada área, o instrumento oferece três opções de resposta: independente, necessita de assistência e dependente. Ao final, é possível verificar em quantas atividades o idoso é dependente^(20)^.
- A Escala de Lawton e Brody foi utilizada para análise da dependência/independência em atividades instrumentais de vida diária (AIVD) em áreas da vida, como a capacidade para usar o telefone, preparar refeições, realizar trabalho doméstico, usar medicamentos e manejo de dinheiro. Esta é uma escala com pontuação final que pode variar de zero a 21 pontos, e podem ser categorizados da seguinte forma: um total de 7 pontos significa dependência total, de 8 a 20 pontos dependência parcial e 21 pontos independência^(21,22)^.
- O Índice de Qualidade do Sono de Pittsburgh (PSQI) foi utilizado para qualificação do sono. É composto por 19 questões autorrelatadas. O instrumento compreende avaliar 7 componentes: qualidade subjetiva do sono, latência do sono, duração do sono, eficiência habitual do sono, alterações do sono, uso de medicamentos para dormir e disfunção diurna do sono. Sua pontuação pode variar de 0 a 21 pontos, podendo ser categorizada em sono de boa qualidade (0 a 4 pontos), sono de má qualidade (5 a 10 pontos) e presença de distúrbios do sono (11 a 21 pontos)^(23)^.
- O *The Addenbrooke’s Cognitive Examination Revised* (ACE-R) foi utilizado para avaliação cognitiva, composto por uma bateria de avaliação cognitiva breve. Compreende cinco domínios cognitivos com pontuações que variam de 0 a 100 pontos, são eles: Atenção e Orientação (pontuação total de 18 pontos); Memória (pontuação total de 26 pontos); Fluência (pontuação total de 14 pontos); Linguagem (pontuação total de 26 pontos) e Visuo-espacial (pontuação total de 16 pontos)^(24)^.
- O Fenótipo de fragilidade proposto por Fried foi utilizado para classificação de fragilidade. O instrumento é composto por cinco elementos: perda de peso não intencional, fadiga, baixa força de preensão palmar, baixo nível de dispêndio calórico e lentidão da marcha. Das cinco características do fenótipo, a presença de três ou mais pode indicar que o estado da pessoa idosa seja categorizado em pré-frágil, frágil ou robusto^(25)^.
- A Escala de Depressão Geriátrica 15 (GDS-15) foi utilizada para rastreio de sintomas depressivos. Trata-se de um questionário com 15 questões, com respostas sim ou não, e escore final que pode variar de zero a 15 pontos, categorizados da seguinte forma: de 0 a 5 pontos, não há indícios de sintomas depressivos; de 6 a 10 pontos, sintomas depressivos leves; e de 11 a 15 pontos, sintomas depressivos severos^(26,27)^.
- A Escala de Estresse Percebido foi utilizada para quantificação do nível de estresse. A escala possui 14 questões com pontuação que varia de zero a 56 pontos. A soma total dos pontos indica que, quanto mais elevado o escore final, maior é o grau de estresse percebido pela pessoa idosa^(28)^.
- O APGAR de família foi utilizado para categorização da funcionalidade familiar. O instrumento é composto por 5 questões que permitem mensurar a satisfação com a família em relação à adaptação, companheirismo, desenvolvimento, afetividade e capacidade resolutiva. Ao final, a pontuação total pode variar entre zero e 20 pontos, e é classificada como: elevada disfunção familiar (0 – 8 pontos), moderada disfunção familiar (9 – 12 pontos) e boa funcionalidade familiar (13 – 20 pontos)^(29)^.
- Para avaliação da qualidade de vida, foram utilizadas as escalas do *World Health Organization Quality of Life*, versão abreviada e versão para idosos (WHOQOL-*bref* e WHOQOL-old). A versão abreviada possui 26 itens que compreendem avaliar quatro domínios: físico, psicológico, relações sociais e meio ambiente, além de avaliar a autopercepção da qualidade de vida e a satisfação com a saúde. Ao final, quanto maior a pontuação, melhor é a percepção da pessoa idosa quanto à qualidade de vida^(30)^. A versão old é composta por 24 questões, que avaliam seis domínios: funcionamento dos sentidos, autonomia, atividades passadas, presentes e futuras, participação social, morte e morrer e intimidade. O escore total varia entre quatro e 20 pontos, e quanto mais elevado o escore final, melhor a percepção da pessoa idosa para a qualidade de vida^(31)^.
- A Escala de Apoio Social do *Medical Outcomes Study* é um instrumento utilizado para análise da disponibilidade de apoio material, emocional, afetivo, informação e interação social positiva. Composto por 19 itens com pontuação que variam entre 20 à100 pontos, onde quanto maior o escore, maior é o nível de apoio social do indivíduo^(32)^. Os instrumentos utilizados para a coleta de dados foram traduzidos e validados para uso no contexto brasileiro.

### Análise dos resultados e estatística

Os dados obtidos foram codificados e digitados em planilha eletrônica e analisados com pacote estatístico Stata versão 13. Foram estimadas distribuições de frequências, médias, desvios padrão, medianas, percentil 25 (p25) e percentil 75 (p75) para as variáveis numéricas e estimadas proporções para as variáveis categóricas. Utilizou-se o teste de Kolmogorov-Smirnov para testar a normalidade das variáveis. As diferenças entre os grupos no caso das variáveis com distribuição normal foram calculadas por meio Teste t e no caso da ausência de normalidade utilizou-se o Teste de Mann-Whitney. Já entre as variáveis categóricas foram utilizados os testes de Qui-quadrado de Pearson e Exato de Fisher. Para a análise de associação, utilizou-se regressão logística múltipla, sendo que a magnitude da associação foi estimada pela razão de chances (*Odds Ratio – OR*) bruta e ajustada. As variáveis que apresentaram valor de p menor que 0,20 na análise univariada foram incluídas no modelo múltiplo por meio do procedimento *stepwise forward*, sendo que ficaram no modelo final as que apresentaram significância estatística (p<0,05) e as variáveis sexo e faixa etária para ajuste. Em todas as análises, utilizou-se um índice de significância de 5%.

## RESULTADOS

A Tabela 1 apresenta as características sociodemográficas dos participantes segundo a má qualidade do sono de forma detalhada. As pessoas idosas dependentes eram, em sua maioria, do sexo feminino (52,54%) e inseridas na faixa etária de 60 a 74 anos (71,19%). Não foram encontradas diferenças estatisticamente significativas entre as características sociodemográficas e a má qualidade do sono. Aproximadamente 76,27% dos participantes apresentaram sono ruim.

**Tabela 1:** Características sociodemográficas de pessoas idosas dependentes segundo a má qualidade do sono. São Carlos, São Paulo, Brasil, 2019-2020

Variável	Total n(%)	Má qualidade do sono			OR(IC95%)
		Não n(%)	Sim n(%)	Valor de *p*	
Sexo					
Masculino	28(47,46)	9(32,14)	19(67,86)	0,149^1^	
Feminino	31(52,54)	5(16,13)	26(83,87)		2,46(0,71-853)
Faixa etária					
60 a 74 anos	42(71,19)	10(23,81)	32(76,19)	1,000^2^	
75 anos ou mais	17(28,81)	4(23,53)	13(76,47)		1,01(0,26-3,82)
Raça/Cor				0,835^2^	
Branca	22(37,29)	6(27,27)	16(72,73)		
Preta	11(18,64)	3(27,27)	8(72,73)		1,00(0,19-5,07)
Parda	25(42,37)	5(20,00)	20(80,00)		1,50(0,38-5,82)
Indígena	0(0,00)	0(0,00)	0(0,00)		-
Amarela	1(1,69)	0(0,00)	1(100,00)		-
Situação conjugal				1,000^2^	
Com companheiro	54(91,53)	13(24,07)	41(75,93)		
Sem companheiro	5(8,47)	1(20,00)	4(80,00)		1,26(1,29-12,37)
Anos de estudo					
≤ 4anos	49(83,05)	11(22,45)	38(77,55)	0,688^2^	
> 4anos	10(16,95)	3(30,00)	7(70,00)		0,67(0,14-3,05)
Religião				0,276^2^	
Católica	33(55,93)	8(24,24)	25(75,76)		
Evangélica	17(28,81)	6(35,29)	11(64,71)		0,58(0,16-2,09)
Outra	7(11,86)	0(0,00)	7(100,00)		-
Não possui	2(3,39)	0(0,00)	2(100,00)		-
Trabalha atualmente					
Não	57(96,61)	14(24,56)	43(75,44)	1,000^2^	
Sim	2(3,39)	0(0,00)	2(100,00)		-
Aposentado(a)				0,179^2^	
Não	8(13,56)	0(0,00)	8(100,00)		
Sim	51(86,44)	14(27,45)	37(72,55)		-
Renda suficiente					
Não	33(55,93)	5(15,15)	28(84,85)	0,081^2^	
Sim	26(44,07)	9(34,62)	17(65,38)		0,33(0,09-1,17)
N° de moradores no domicílio					
≤2pessoas	35(59,32)	7(20,00)	28(80,00)	0,416^1^	
>2pessoas	24(40,68)	7(29,17)	17(70,83)		0,60(0,18-2,03)
Funcionalidade familiar*					
Funcional	38(67,86)	7(18,42)	31(81,58)	0,217^1^	
Disfuncional	18(32,14)	6(33,33)	12(66,67)		0,45(0,12-1,62)
Total	59(100,00)	14(23,73)	45(76,27)		

^1^Teste Qui-quadrado de Pearson; ^2^Teste Exato de Fisher: *alguns participantes não responderam; OR: Odds ratio.

Não foram encontradas diferenças estatisticamente significantes entre as características sociodemográficas e a má qualidade do sono.

A Tabela 2 apresenta de forma detalhada as características de saúde das pessoas idosas dependentes de acordo com a má qualidade do sono.

**Tabela 2:** Características de saúde das pessoas idosas dependentes segundo a má qualidade do sono. São Carlos, São Paulo, Brasil, 2019-2020

Variável	Total n(%)	Má qualidade do sono			OR(IC95%)
		Não n(%)	Sim n(%)	Valor de *p*	
Plano de Saúde					
Não	53(89,83)	11(20,75)	42(79,25)	0,139^2^	
Sim	6(10,17)	3(50,00)	3(50,00)		0,26(0,04-1,48)
Queda no último ano					
Não	41(69,49)	12(29,27)	29(70,73)	0,189^2^	
Sim	18(30,51)	2(11,11)	16(88,89)		3,31(0,65-16,67)
Internação no último ano				0,425^2^	
Não	49(83,05)	13(26,53)	36(73,47)		
Sim	10(16,95)	1(10,00)	9(90,00)		3,25(0,37-28,21)
IMC*					
Baixo peso	6(10,71)	1(16,67)	5(83,33)	0,905^2^	
Eutrófico	17(30,36)	5(29,41)	12(70,59)		0,48(0,04-5,22)
Sobrepeso	33(58,93)	8(24,24)	25(75,76)		0,62(0,63-6,17)
Atividade física					
Não	41(69,49)	7(17,07)	34(82,93)	0,070^1^	
Sim	18(30,51)	7(38,89)	11(61,11)		0,32(0,09-1,12)
Dor*					
Não	13(22,81)	3(23,08)	10(76,92)	1,000^2^	
Sim	44(77,19)	10(22,73)	34(77,27)		1,02(0,23-4,43)
Multimorbidade					
Não	1(1,69)	1(100,00)	0(0,00)	0,237^2^	
Sim	58(98,31)	13(22,41)	45(77,59)		-
Polifarmácia*					
Não	28(48,28)	10(35,71)	18(64,29)	0,067^2^	
Sim	30(51,72)	4(13,33)	26(86,67)		3,61(0,97-13,33)
Fragilidade*					
Não	29(50,88)	10(34,48)	19(65,52)	0,123^2^	
Sim	28(49,12)	4(14,29)	24(85,71)		
Sintomas depressivos*					
Não	28(50,00)	9(32,14)	19(67,86)	0,205^2^	
Sim	28(50,00)	4(14,29)	24(85,71)		2,84(0,75-10,66)
Declínio Cognitivo					
Não	11(18,64)	1(9,09)	10(90,91)	0,269^2^	
Sim	48(81,36)	13(27,08)	35(72,92)		0,26(0,03-2,31)
ABVD					
Independente	30(50,85)	7(23,33)	23(76,67)	0,942^1^	
Dependente	29(49,15)	7(24,14)	22(75,86)		0,95(0,28-3,17)
AIVD					
Independente	1(1,69)	0(0,00)	1(100,00)	1,000^2^	
Dependente	58(98,31)	14(24,14)	44(75,86)		-
Tabagismo					
Não	51(86,44)	14(27,45)	37(72,55)	0,179^2^	
Sim	8(13,56)	0(0,00)	8(100,00)		-
Etilismo					
Não	51(86,44)	12(23,53)	39(76,47)	1,000^2^	
Sim	8(13,56)	2(25,00)	6(75,00)		0,92(0,16-5,18)

^1^Teste Qui-quadrado de Pearson; ^2^Teste Exato de Fisher; *alguns participantes não responderam; OR: Odds ratio; IMC: Índice de Massa Corporal; ABVD: Atividades Básicas da Vida Diária; AIVD: Atividades Instrumentais da Vida Diária.

Não foram encontradas diferenças estatisticamente significantes entre as características de saúde e a má qualidade do sono.

A maioria das pessoas idosas consumia café (84,75%) e não fazia uso de chá verde (76,27%), chá mate (71,19%), chá preto (91,53%), chocolate (72,88%), gengibre (77,97%), pimentas (64,41%) e nem guaraná (98,31%). Não foram encontradas diferenças estatisticamente significantes entre os grupos (boa/má qualidade do sono) em relação ao consumo destas bebidas e alimentos.

A Tabela 3 apresenta a relação entre as variáveis estresse, qualidade de vida e apoio social segundo a má qualidade do sono de pessoas idosas dependentes.

**Tabela 3:** Análise comparativa entre as variáveis estresse, qualidade de vida e apoio social segundo a má qualidade do sono de pessoas idosas dependentes. São Carlos, São Paulo, Brasil, 2019-2020

Variável	Má qualidade do sono				Valor de *p*
	Não		Sim		
	Média (dp)	Mediana (p25;p75)	Média (dp)	Mediana (p25;p75)	
Estresse	18,00 (10,16)	17,00 (13;27)	24,86 (10,93)	24,00 (19;33)	0,041^1^
Qualidade de vida	69,40 (22,02)	72,08 (64,16;84,16)	66,00 (17,35)	68,33 (64,16;76,66)	0,268^2^
Apoio Material	85,71 (27,44)	100 (80;100)	87,44 (23,27)	100 (85;100)	0,884^2^
Apoio Afetivo	86,19 (26,20)	96,66 (80;100)	84,59 (26,62)	100 (86,66;100)	0,845^2^
Apoio Emocional	75,71 (26,80)	80 (65;100)	76,55 (27,54)	90 (65;100)	0,758^2^
Interação Social Positiva	77,85 (26,21)	80 (70;100)	71,11 (27,77)	75 (60;100)	0,361^2^
Informação	78,92 (26,54)	85 (70;100)	74,22 (28,02)	85 (50;100)	0,683^2^

*^1^ Teste t; ^2^ Teste de Mann-Whitney.*

Observou-se relação estatisticamente significativa entre estresse e má qualidade do sono. Pessoas idosas dependentes com má qualidade do sono apresentaram maiores escores de estresse quando comparadas àquelas com sono bom.

No modelo de regressão logística múltiplo o estresse (OR=1,12; IC95%=1,02-1,22) e o uso de polifarmácia (OR=7,39; IC95%=1,2244,73) aumentaram as chances de má qualidade do sono em pessoas idosas dependentes, enquanto a realização de atividade física diminuiu tais chances (OR=0,15; IC95%=0,02-0,79), independente do sexo e da faixa etária, conforme Tabela 4. A Tabela 4 apresenta os fatores associados à má qualidade do sono.

**Tabela 4:** Fatores associados à má qualidade do sono de pessoas idosas dependentes. São Carlos, São Paulo, Brasil, 2019-2020

	OR_aj_^1^	Valor de *p*	IC95%
Estresse	1,12	0,017	1,02-1,22
Atividade Física			
Não	1,00		
Sim	0,15	0,026	0,02-0,79
Polifarmácia			
Não	1,00		
Sim	7,39	0,029	1,22-44,73
Sexo			
Masculino	1,00		
Feminino	0,77	0,756	0,15-3,79
Faixa etária			
60 a 74 anos	1,00		
75 anos ou mais	1,38	0,688	0,28-6,74

*^1^OR_aj_=Odds ratio ajustada; IC95%: Intervalo de confiança em 95%.*

## DISCUSSÃO

Esse estudo identificou os fatores associados à má qualidade do sono de pessoas idosas dependentes inseridas em regiões de vulnerabilidade social. Fatores como estresse e uso de polifarmácia aumentaram as chances de má qualidade do sono, enquanto a realização de atividade física diminuiu tais chances, independentemente do sexo e da faixa etária.

No presente estudo, houve predomínio de má qualidade do sono entre as pessoas idosas dependentes. Pesquisas nacionais e internacionais corroboraram esses achados, as quais também identificaram elevadas proporções de queixas relativas ao sono noturno^(33,34,35)^.

Evidências na literatura apontam que algumas alterações que acontecem no organismo dos indivíduos ao longo do processo de envelhecimento são capazes de interferir tanto na quantidade quanto na qualidade do sono noturno. Fatores como redução da melatonina, modificações no ritmo circadiano, disfunções neuroendócrinas, comorbidades médicas, diminuição do envolvimento social e perturbações ambientais contribuem para o prejuízo do sono noturno^(34,35,36,37)^. Ademais, as evidências demonstram ainda que fatores familiares e condições sociais podem afetar a qualidade do sono^(38)^.

No presente estudo, houve associação entre má qualidade do sono e estresse. Um estudo realizado na Índia, com o objetivo de identificar a prevalência de má qualidade do sono e seus fatores associados, observou 180 pessoas idosas. Como resultado, obteve que 68,9% da amostra apresentava má qualidade do sono e os fatores associados ao sono ruim foram depressão, ansiedade e não possuir companheiro^(35)^.

Corroborando esses achados, uma pesquisa realizada nos EUA com 4.201 pessoas com 65 anos de idade e mais identificou que aqueles altamente estressados tiveram quase cinco vezes mais chances de apresentar sono de má qualidade quando comparados àqueles com baixo nível de estresse^(12)^.

Pesquisadores chineses realizaram um estudo com pessoas idosas e constataram uma correlação significativa entre qualidade do sono e sofrimento psicológico, inclusive estresse. Observaram ainda que a má qualidade do sono pode piorar o humor e aumentar a ansiedade, gerando altos níveis de estresse ao longo do dia^(39)^.

Para aqueles com prejuízo funcional dependentes em atividades diárias, foi possível observar altos níveis de ansiedade e depressão, podendo culminar em intenso estresse e sono de má qualidade^(39)^. Muitas vezes, por não conseguirem atender suas necessidades independentemente, esses indivíduos podem se sentir inúteis, o que afeta a saúde mental e prejudica o sono noturno^(39)^. Embora se refira a uma província, compreendendo 11 cidades da China Central, é possível que este raciocínio possa explicar, em parte, a associação identificada entre estresse e sono de má qualidade no presente estudo, tendo em vista que houve maior prevalência de má qualidade do sono entre pessoas idosas dependentes, tanto para ABVD quanto para AIVD. Por outro lado, experimentar um sono noturno ruim (seja pela dificuldade em iniciar e manter o sono, seja pela curta duração desse sono) pode trazer prejuízos para o dia subsequente, como piora do humor, aumento da ansiedade e fadiga diurna, o que poderia gerar estresse^(39)^.

Na tentativa de reduzir o estresse e, consequentemente, melhorar a qualidade do sono noturno, um ensaio clínico randomizado com pessoas idosas testou os efeitos do mindfulness durante 8 semanas e acompanhamento por 6 meses. Como resultado, obtiveram que a redução de estresse baseada em mindfulness foi efetiva, capaz de melhorar a qualidade do sono com moderados a altos níveis de distúrbios do sono. Concluíram que intervenções mente-corpo são relevantes na abordagem de fatores associados à má qualidade do sono^(40)^.

Diante de condições geradoras de estresse, pesquisadores afirmam que o cortisol é liberado para que haja adaptação às demandas do ambiente. Entretanto, a secreção prolongada de hormônios do estresse pode trazer prejuízos ao indivíduo, em decorrência do aumento da inflamação crônica, da redução da imunidade, do desequilíbrio homeostático e da inibição da liberação de melatonina, o que prejudicaria o sono noturno^(41)^.

Dentre as pessoas idosas dependentes, o uso de polifarmácia aumentou cerca de 6,39 vezes as chances de má qualidade do sono. O número de medicamentos em uso também foi relacionado a queixas de sono em recentes estudos de revisão identificados na literatura internacional^(42)^.

Pesquisadores apontam que vários mecanismos podem estar envolvidos na explicação do comprometimento do sono noturno em pessoas idosas que fazem uso de polifarmácia em virtude da influência exercida sobre os receptores e neurotransmissores. A sonolência diurna (com o uso de anti-histamínicos, anticonvulsivantes, opiáceos, por exemplo), o efeito estimulante (com o uso de agonistas beta, pseudoefedrina, selegilina, por exemplo), o potencial de agravar distúrbios do sono já existentes (com o uso de antidepressivos, opiáceos, benzodiazepínicos, por exemplo), a capacidade de prejudicar a arquitetura do sono mediante a redução de secreção de melatonina (betabloqueadores, por exemplo), a interação medicamento-medicamento e/ ou medicamento-doença que pode culminar em incômodo ao sono noturno (nictúria, tosse, hipoglicemia, por exemplo) e os efeitos colaterais são fatores que podem elucidar essa relação encontrada entre polifarmácia e sono ruim^(42,43)^.

Os resultados da presente pesquisa mostraram que a atividade física foi identificada como fator protetor em relação ao sono ruim. Achados convergentes foram identificados na literatura^(44,45)^. Uma revisão de literatura realizada com ensaios clínicos randomizados, quase-experimentais e descritivos na Espanha observou que o exercício físico ofereceu efeitos positivos (p<0,05) para melhoria da qualidade do sono, especialmente em pessoas com idade superior a 55 anos e mulheres^(44)^.

Com o objetivo de investigar o efeito do exercício físico na qualidade do sono e nas citocinas inflamatórias em pessoas idosas, um ensaio clínico randomizado foi realizado com 50 participantes, os quais foram divididos aleatoriamente em dois grupos (A: grupo intervenção com exercícios aeróbicos supervisionados três vezes por semana ao longo de seis meses e B: grupo controle, sem intervenção que seguiu o estilo de vida normalmente). Como resultado, obtiveram que o treino aeróbico reduziu os níveis de citocinas pró-inflamatórias e melhorou a qualidade do sono noturno^(45)^.

Embora a atividade física e o exercício físico não sejam sinônimos, percebe-se que tais termos têm sido utilizados de maneira intercambiável na literatura, a qual têm mostrado resultados promissores na qualidade do sono quando são realizados de maneira regular^(44)^.

Um recente estudo de revisão, mostrou que o exercício físico exerce um efeito benéfico sobre a qualidade do sono noturno de pessoas idosas comunitárias. Os resultados mostraram que exercícios aeróbicos, de baixa a moderada intensidade, durante 30 minutos diários, com frequência de três a cinco vezes por semana são capazes de repercutir em sono noturno de boa qualidade. Concluíram que a atividade física aliada à higiene do sono atua como um fator protetor aos distúrbios do sono^(44)^.

Esses pesquisadores apontaram que o mecanismo relacionado ao efeito positivo dos exercícios físicos sobre a qualidade do sono ainda não está claramente definido. Acredita-se que a secreção de endorfina e o aumento do consumo de energia facilitariam um sono tranquilo e, consequentemente, a recuperação do corpo. Ademais, a redução do estado inflamatório de baixo grau também refletiria efeitos benéficos sobre a qualidade do sono^(45)^.

Outros estudiosos apontam que a prática regular de atividade física é capaz de melhorar a saúde das pessoas idosas de maneira generalizada, sendo notório o impacto benéfico da adoção de um estilo de vida saudável. O exercício físico pode fortalecer as habilidades funcionais bem como postergar ou neutralizar o declínio funcional, contribuindo para melhoria da qualidade de vida e redução do sofrimento psicológico^(39)^.

Diante do contexto de vulnerabilidade social, caracterizada pelos escassos recursos assistências, sejam de natureza social ou de saúde, a assistência prestada no âmbito da APS é fundamental para que seja ofertado uma assistência baseada em ações assertivas e qualificadas, fundamentalmente pautadas na compreensão das necessidades da pessoa idosa, considerando as especificidades do envelhecimento e sobretudo centrado em cuidados integrais ao indivíduo, família e coletividade^(46)^.

### Limitações do estudo

Os resultados deste estudo estão sujeitos a limitações. Diante do delineamento transversal, da amostra reduzida e específica, existem limitações em termos de generalização e inferência de causalidade. Além disso, a capacidade explicativa dos modelos utilizados neste estudo foi de baixa a moderada, mesmo após incluir um número significativo de variáveis potencialmente explicativas. Pesquisas futuras, principalmente de delineamento longitudinal, são necessárias para aprofundar a temática.

### Contribuições para a Enfermagem

Os achados do presente estudo têm implicações potenciais para o desenvolvimento de políticas e intervenções que podem contribuir no âmbito da atenção à saúde, fomentando recomendações para reduzir a má qualidade do sono nessa população. Além disso, os resultados podem orientar a projeção de ações de enfermagem e cuidados de saúde, considerando os fatores associados à má qualidade do sono e as variáveis que podem minimizá-la, bem como o contexto social. Isso é importante porque a má qualidade do sono pode prejudicar a saúde, a qualidade de vida e o bem-estar da pessoa idosa.

## CONCLUSÃO

A hipótese foi parcialmente confirmada. O estresse e o uso de polifarmácia aumentaram as chances de má qualidade do sono e, por outro lado, a prática de atividade física reduziu essa chance.
